# External release of entropy by synchronized movements of local secondary structures drives folding of a small, disulfide-bonded protein

**DOI:** 10.1371/journal.pone.0198276

**Published:** 2018-06-12

**Authors:** Atsushi Sato, Andre Menez

**Affiliations:** 1 Department of Information Science, Faculty of Liberal Arts, Tohoku Gakuin University, Sendai, Japan; 2 CEA/DSV/DIEP, Gif sur Yvette, Paris, France; The University of Texas at El Paso, UNITED STATES

## Abstract

A crucial mechanism to the formation of native, fully functional, 3D structures from local secondary structures is unraveled in this study. Through the introduction of various amino acid substitutions at four canonical β-turns in a three-fingered protein, Toxin α from *Naja nigricollis*, we found that the release of internal entropy to the external environment through the globally synchronized movements of local substructures plays a crucial role. Throughout the folding process, the folding species were saturated with internal entropy so that intermediates accumulated at the equilibrium state. Their relief from the equilibrium state was accomplished by the formation of a critical disulfide bridge, which could guide the synchronized movement of one of the peripheral secondary structure. This secondary structure collided with a core central structure, which flanked another peripheral secondary structure. This collision displaced the internal thermal fluctuations from the first peripheral structure to the second peripheral structure, where the displaced thermal fluctuations were ultimately released as entropy. Two protein folding processes that acted in succession were identified as the means to establish the flow of thermal fluctuations. The first process was the time-consuming assembly process, where stochastic combinations of colliding, native-like, secondary structures provided candidate structures for the folded protein. The second process was the activation process to establish the global mutual relationships of the native protein in the selected candidate. This activation process was initiated and propagated by a positive feedback process between efficient entropy release and well-packed local structures, which moved in synchronization. The molecular mechanism suggested by this experiment was assessed with a well-defined 3D structure of erabutoxin b because one of the turns that played a critical role in folding was shared with erabutoxin b.

## Introduction

**Fig 1:**
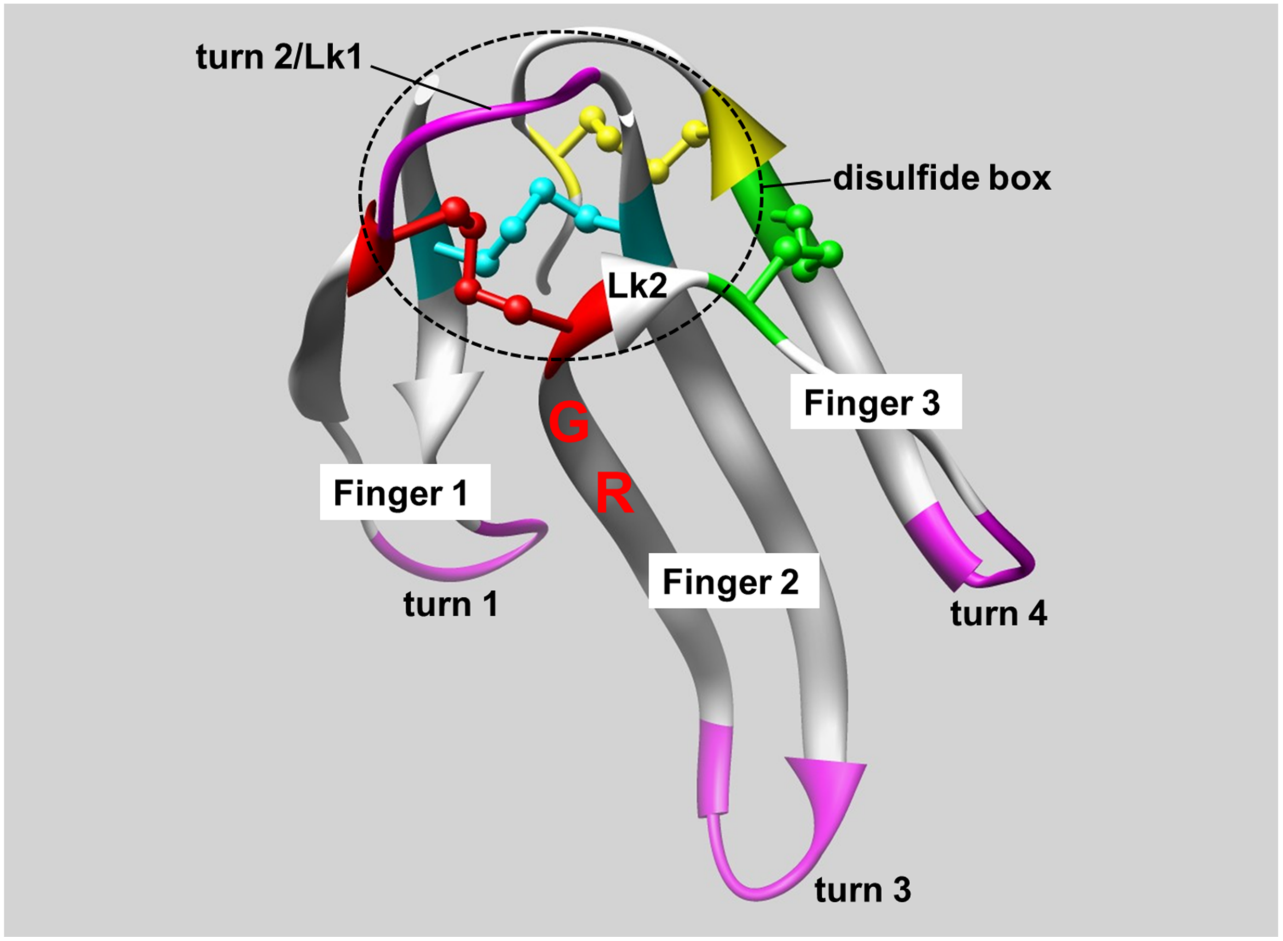
Four disulfide bridges and a β pleated sheet characterize the TFPD. The structure of erabutoxin b from *Laticauda semifasciata* (3EBX.pdb [2]). The Turn 2 in Lk1 is unique to erabutoxin b, while the rest of the structure is essentially shared with Toxin α [2,5]. Tox62 is a chimeric protein used in this study with the amino acid sequence LECHNQQSSQPPTTKTCSPGETNCYKKVWRDHRGTIIERGCGCPTVKPGIKLNCCTTDKCNN, using the underlined SPGE motif that forms turn 2 in erabutoxin b and the rest of the sequence coming from Toxin α. A previous study had shown that this SPGE motif in Tox62 reduced the folding rate [8]. Labels R and G represent R39 and G40, which were predicted to form a turn (see text). Four disulfide bridges, 3–24 (cyan), 17–41 (red), 43–54 (green), and 55–60 (yellow), are shown with sulfur atoms. The four turns targeted for mutation are shown in purple (QSSQ (7–10, turn 1), SPGE (18–21, turn 2), DHRG (31–34, turn 3), and PGIK (48–51, turn 4)) and were replaced with other sequences to prepare mutants. The fifth turn in the structure (residue 57–60) was not colored because it was not a major subject of this study. Lk1 and Lk2 are the peptide chains of C17-SPGETN-C24 and C41-G-C43, respectively. They link the N-terminal and C-terminal structural domains.

The three-fingered protein domain (TFPD) is a small, functional module composed of 60 to 90 amino acid residues that form three successive fingers [1–5]. A cluster of three disulfide bonds form “disulfide box” that is a rigid core structure. This core structure and another nearby disulfide bond hold the fingers together (Fig 1). The structure of the TFPD was initially established in erabutoxin b [6,7], from *Laticauda semifasciata*, which binds to the nicotinic acetylcholine receptor (nAChR). Activins, bone morphogenetic proteins, inhibins, Urokinase/plasminogen activator receptors and others have been found to have from one to three TFPDs [1].

The significant structural attributes of the TFPD fold are as follows: (1) The N-terminus half of the domain is primarily stabilized by inter-strand hydrogen bonds to form rigid β-pleated sheets. (2) The C-terminus half of the domain, primarily finger 3, forms a flexible, oil drop-like structure from clusters of hydrophobic side chains. (3) The typical β hairpin structure of finger 2 forms a β pleated sheet with finger 3. (4) Denatured, fully oxidized cobrotoxin refolds rapidly within 250 ms [9]. (5) Three disulfide bonds are located close to each other, and an invariant asparagine residue near the C-terminus (N61 in erabutoxin b) is essential to hold their relative orientation [1]. These three disulfide bonds are essentially buried in the folded state, while the fourth disulfide bond (S43-S54 in erabutoxin b) is solvent-exposed [10]. (6) A refolding study of entirely reduced and denatured Toxin α and its mutant Tox62 has identified two folding intermediates, the C form (des [43–54]) and D form (des [17–41])[8,11,12]. The deletion of one or two residues in Lk1 (Fig 1) accelerated folding. This result has been understood to correlate with the conformational variability of the intermediates [8,10].

The slow folding and the well-defined intermediates expectedly allow a detailed investigation of the critical processes, such as complex structural transition steps, involved in protein folding. The results should also provide some clues for evaluating the various folding models [13–21]. This study was therefore anticipated to dissect the complex structural transition process. In this study, mutations were introduced in the Tox62 sequence to mimic antagonizing inhibitors, which have been used successfully to visualize complex physiological processes. Turn sequences were selected as the targets of mutation because the primary role of the folding-active turns is to drive the process towards the transition state [22].

As had been expected, using a tetra-peptide with a high turn-forming propensity at the active turns, such as YNGK, accelerated the fourth disulfide bond formation, while the native-like flexibility of the resulting four-disulfide bond species was unexpectedly severely impaired. At the passive turns [22], reversed contributions of the mutations were observed. These results suggested that a rigid turn structure at the active turn, such as that of YNGK, successfully divided the folding process into two distinct processes, the assembly process and the activation process. After the time-consuming trial and error in the assembly process, efficient entropy release was established by the molecular-wide global synchronization during the activation process.

## Materials and methods

### Design of proteins

Turn-forming propensities were calculated according to Hutchinson and Thornton [23] (Table 1). The hydrophobicity indices of Tossi *et al*. [24] were used to calculate the average hydrophobicity for each turn. For its very high type I turn-forming propensity (398), as well as its relatively high type II turn-forming propensity (15), YNGK was expected to form a rigid beta hairpin structure, which is common among the structures of sequences flanking any type I, I’, II, or II’ turns. QAAQ and FDGQ were expected to work as references for YNGK; QAAQ for its low turn-forming propensity and similar hydrophobicity, and FDGQ for its or high turn-forming propensity and higher hydrophobicity. The deletion of two residues at turn 2, to yield PG, reportedly accelerated folding [8].

**Table 1:** Turn-forming propensities and hydrophobicities of turn sequences.

Turn sequences	Native/Mutant	Location of turns	Hydro-phobicity[24]	Turn-forming propensity of Hutchinson & Thornton [23]				
				type I	type II	type VIII	type I’	type II’
QSSQ	Native	1	-5.16	0.8	0.5	0.6	0.3	1.8
SPGE	Native	2	-3.80	3.3	47.9	0.5	0.0	0.0
CPGE	Native	2	-3.30	3.4	7.1	0.4	0.0	0.0
DHRG	Native	3	-6.12	2.8	0.0	0.2	0.3	0.0
KPGI	Native	4	-0.95	1.1	53.0	0.1	0.0	0.0
YNGK	Mutant	1, 2, 3, and 4	-4.22	0.3	15.0	0.1	398.0	0.9
QAAQ	Mutant	1, 2, 3, and 4	-3.55	0.4	0.3	0.4	0.1	0.7
CPGT	Mutant	2	-2.17	3.7	6.6	1.1	0.0	0.0
FDGQ	Mutant	3	-1.67	0.3	16.1	0.2	42.2	1.1

The types of each turn in the respective native structures are highlighted with thick boxes. Turns 2 and 4 are suggested to be active turns by their high turn-forming propensities in native sequences. SPGE and CPGE are the turn 2 sequences in erabutoxin b and Toxin α, respectively.

### Prediction of turns

Thirty-two amino acid sequences of snake toxins [1], including short and long neurotoxins, cardiotoxins, and fasciculins, were analyzed for their turn forming probabilities using two different prediction methods. One was SVMturn [25], a support vector, machine-based method, and the other was SOPMA [26], which made use of multiple alignments of homologous sequences. The prediction for each residue to form a turn was scored as 0, 1, or 2, representing the number of methods that predicted a turn at each position. These scores were aligned based on the DSSP parameters [27,28] for secondary structures (PDB) and summarized for each position.

### Preparation of Tox62 and its mutant proteins

Tox62 and its mutants were prepared by either a peptide synthesizer (Applied Biosystems, model 431) or by genetic engineering, as previously described [29]. Synthesized products were oxidized overnight in an ambient temperature at a protein concentration of 0.1 mg/ml in the presence of 3 mM glutathione sulfide (GSH), 0.3 mM of glutathione disulfide (GSSG) and 1 mM ethylenediaminetetraacetic acid (EDTA) in 0.1 M tris(hydroxymethyl)aminomethane hydrochloride (Tris-HCl), pH 7.6. The reaction mixture was subjected to preparative RP-HPLC (reverse-phase high-performance liquid chromatography), and folded species with four disulfide bridges were collected and lyophilized.

### *In vitro* refolding and analysis of the products on RP-HPLC

Prior to the refolding reaction, sample proteins were reduced and denatured with a 50-fold molar excess of dithiothreitol (DTT) and 6 M guanidine hydrochloride (GuHCl) in 0.1 M Tris-HCl, 1 mM EDTA, pH 8.5 for 2 hours at 37°C. Four volumes of 0.1% trifluoroacetic acid (TFA) was added to dilute the reaction mixture, the acidified sample was applied to an RP-HPLC column, and a denatured and reduced protein fraction was collected, with a linear gradient of 0.1% TFA to 36% acetonitrile-0.1% TFA, and lyophilized. Sample protein solution was prepared with 100 μl of chilled 0.01% acetic acid, which had been prepared with degassed and argon bubbled (20 min) MilliQ water. The protein concentration was adjusted to 50 μM using calculated molar absorption coefficients. The refolding reaction was performed using 15 μM of protein, 3 mM GSH/0.3 mM GSSG, and 1 mM EDTA in 0.1 M Tris-HCl, pH 7.5 at 25°C under an argon atmosphere. Aliquots of samples were withdrawn at each time point and added to 0.1 volume of 3 M HCl to stop the disulfide bridge exchange [30]. Samples were frozen with liquid nitrogen and stored at -80°C until analysis. This protocol was followed in parallel for both the sample protein and Tox62 as the control. Elution of the refolding reaction mixture from a reverse phase HPLC column (Chromolith Performance RP-18e, 100–4.6 mm (MERCK)) was performed with a linear gradient of 12–30% acetonitrile in 0.1%TFA over 8 min, at a flow rate of 2 ml/min, at ambient temperature. The effluent was monitored for absorbance at 214 nm.

### Blocking of free sulfhydryl groups by monoiodoacetamide

Tox62 (1.41 mg) was denatured and reduced in 7 ml of buffer containing 50-fold molar excess of dithiothreitol (DTT), 6 M GuHCl, 0.1 M Tris-HCl, pH 8.5, and 1 mM EDTA at 37°C for 1 h, as for denaturation and reduction reactions in previous refolding experiments. The lyophilized product was refolded for 70 min in 10.6 ml of refolding mixture (0.1 M Tris-HCl, 1 mM EDTA, pH 7.5, 3 mM GSH / 0.3 mM GSSG, and 15 μM of protein) under argon at 25°C. The reaction was stopped by the addition of 10.6 ml of 2.2 M monoiodoacetamide (IAM) solution. After 30 seconds of reaction, the reaction mixture was acidified with 500 μl of 20% TFA. Separation of IAM-blocked C and D forms was performed on RP-HPLC (250 x 10 mm 10-micron, Phenomenex Jupiter C18). Collected fractions were further subjected to re-chromatography to complete purification. The molar extinction coefficient used was 9,000 at 280 nm.

### CD spectrometry

Circular dichroism spectra were monitored at 20°C from 186 to 260 nm, using quartz cells of 0.1 cm path length and a Jobin Yvon CD6 spectropolarimeter. Data were collected with 10 μM HPLC preparations, in 2 mM phosphate buffer at pH 7.0, of N, C and D forms in the absence of a sulfur reduction/oxidation agent. Immediately after the data collection, samples of the C form and the D form were acidified using 0.1 volume of 3 M HCl and applied to HPLC to correct iteratively for the oxidation product(s) present at data collection.

### Binding assay to nAChR

The binding property to nAChR was assessed as previously described [31].

### Calculation of relative rate constants

Relative rate constants were calculated using Kintecus [32], with the fitting/optimization algorithm of Nelder & Mead [33]. The relative amounts of each folding species were normalized to satisfy U+U2+C+D+N = 1, where U denotes the entirely reduced species and U2 denotes a mixture of various species that directly or indirectly provide C or D forms [12]. The early emergence of a two-disulfide species (2S) that persist at detectable amounts had previously been reported for erabutoxin b, where the amino acid sequence of Lk1 was invariant with that of Tox62, and its folding rate was as slow as that for Tox62 [8]. A significant component of the U2 form could thus be the 2S species, with S3-S24 and S55-S60, but a large excess of reduced and oxidized glutathione should have supported the rapid intramolecular and intermolecular exchange of the disulfide bonds in the unfolded intermediates. Transient rapid equilibrium was thus expected among the various members of the U2 form. Therefore, as a practical approximation, the initial reaction in the folding process was represented by the reaction of U to U2. The relative rate constant of this reaction was set to 1.8 (in arbitrary units), based on the fact that the fully unfolded species, U, was not detected at 15 min after the initiation of the reaction. Fitting started with U as 1.0 and the others as 0.0 at zero time. Ten sets of relative rate constants were obtained from ten independent folding experiments for Tox62. The elimination of some reactions was made based on the following criteria. (1) Essential reactions are those with large rate constants, and their absence results in the severe impairment of fitting. (2) Candidate reactions to be removed from the final list increase the standard deviations of the rate constants of the principal reactions. The logic of this criterion was the following. Inevitably, experimental random error was present in the observed relative abundance of N, C, and D forms. The fitting was performed ten times separately for each data from a single folding experiment. The ten calculated rate constants for a particular principal reaction, such as a1, a2, …, and a10, were thus inevitably fluctuated by such error. Additional artificial reactions should improve single fitting by increasing the opportunity of mutual compensation to reduce the differences between observed and calculated values. However, this results in more significant deviations among the ten calculated values of the rate constants of a particular principal reaction, such as a1, a2, …, and a10, due to the random property of the error in the observed values. The candidate reactions were therefore examined one by one in the presence of the principal reactions. The significance of the increase in standard deviation was then estimated using the Student’s t-test for pairs of standard deviations for each rate constant of the principal reactions.

### Simulation of the swirling oscillation of atoms

The molecular-wide stochastic process should search for the efficient release of entropy to the external environment. It is therefore worth attempting to investigate this possibility by simulation. Due to the asymmetric configuration and flexible torsion angles of L-amino acids, repetitive intra-molecular movements by repulsive and attractive mutual interactions would drive the unidirectional swirling oscillations of local structures. Trajectories of the synchronized atoms were simulated, where atoms in local structures swirl by coordinated back and forth rotations along two axes that are perpendicular to each other. The synchronized movements of flexible turns, and other flexible local structures, should permit movements that appear similar to a single-fulcrum movement. All of these axes are found in the “disulfide box” (Fig 1), where the structure was reinforced with two disulfide bonds and various hydrogen bonding, as well as by the adjacent pleated sheet. The angle of back and forth rotation in this simulation was ±10 degrees in 5ebx.pdb, except for some oscillations as described below. One cycle of oscillation was simulated for 16 points (every 22.5 degrees over 360 degrees). The coordinates of the swirling atoms were calculated by the successive application of rotation matrices, which were derived from the respective coordinates of the atoms forming the axes. Some delay in the phase of rotation was postulated to account for the situations where movements of some local structures were forced by the movements of other local structures. For the coordinates of the alpha carbon of Q10 (Q10CA) in the oscillating finger 1, one of the axes of rotation was formed by two hydrogen bonds between the backbone atoms of C3 and K15. This axis was essentially perpendicular to a plane formed by the simulated trajectory of the alpha carbon of C41 (C41CA), as well as to the axis formed by the CA-CB bonding of C3. For the axes which were oriented in parallel to this CA-CB bonding of C3, the phase and amplitude of the oscillation is shared among them so that rigid structure of the “disulfide box” (Fig 1) was conserved. For the calculation of the coordinates of Q10CA, the angle of oscillation along the C3-K15 axis was calculated on a plane which was perpendicular to the rotation axis. The coordinates of C17CA and C41CA were projected on that plane to find that the atomic distance between C17CA and C41CA on that plane was 5.89 Å, and the distance between this rotation axis and C17CA was 4.25 Å. With these values and the respective distances of C41CA from the rotation axis, the angle formed by C17CA, the rotation axis, and C41CA was obtained to estimate the coordinates of Q10CA.

### Other methods

Electrospray mass spectrometry (ES-MS) was performed by Atheris Laboratories, Geneva, Switzerland. The hydrophobicities of the HPLC samples were calculated as a summation of the hydrophobicities of four tetra peptides at turns. Chimera [34] was used to draw the molecular structures.

## Results

### Evaluation of turn-forming properties

The propensities [23] to form β turns, shown in Table 1, suggested that turns 2 and 4 were “folding-active” [22], while turns 1 and 3 were “folding-passive” [22], in both toxin α and erabutoxin b.

Two independent methods [25,26] were used to predict turns for each amino acid residue in 32 snake toxin sequences. R39 and G40 had high potentials to form a turn, in spite of the fact that 93% of the 64 residues at these positions were assigned as an extended form by DSSP [27,28]. The summed scores of turn predictions for the 39th (R in Tox62) and 40th (G in Tox62) residues were 53 and 47, respectively. These values were comparable to those of other highly scored residues, such as P19 (score of 51) and G20 (score of 51) at turn 2, which was suggested to be “folding-active”. R39 in Tox62 was replaced with L, K, Q and F in7, 4, 1, and 1 toxin sequence(s) out of 32, respectively, while G40 was invariant. The rate of predicted turns for LG dropped to 29% of the rate for R (or K) G. The third highest prediction scores were 20 and 25 for H30 and R31 at turn 3, respectively. The fourth-most top scores were 19 and 18 for P48 and G49 at turn 4, respectively, where amino acid sequences were found to be rather diverse among the toxins. The normalized Shannon’s entropy [35] ranged from 0.61 to 0.69 at turn 4. Turn 4 might not have to work as a folding active turn in some TFPDs. The assignment of turns by DSSP was somewhat ambiguous for turn 1, and the prediction scores for turn 1 were much lower than the others (4 and 5 for the 8th and 9th residues, respectively, were the highest scores).

### Characterization of the D form

**Fig 2:**
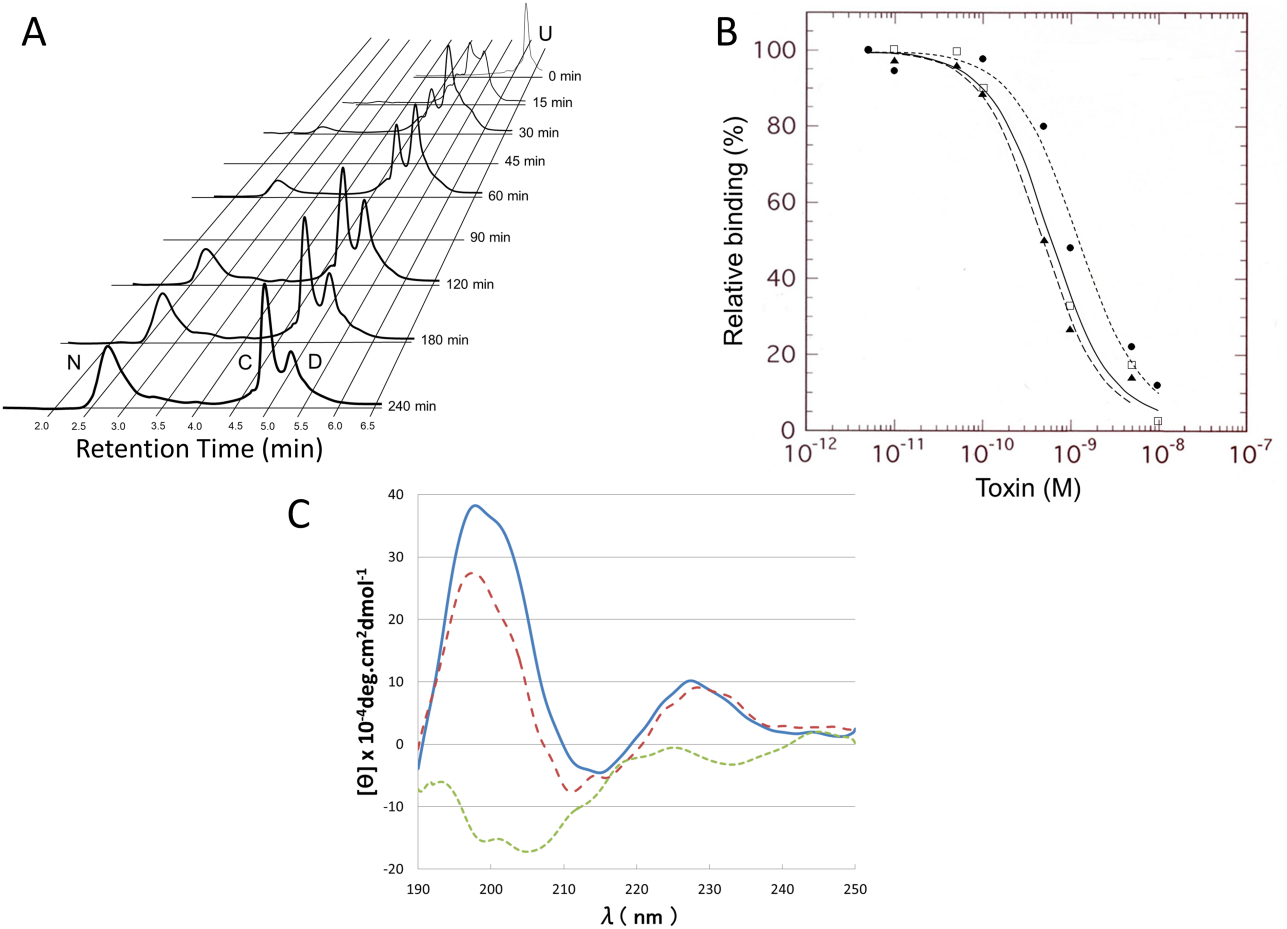
The C form was more native-like than the D form. (A) HPLC elution profiles of the refolding reaction mixture of Tox62. The N, D, and C forms were eluted at 2.7 min, 4.9 min, and 5.3 min, respectively. Elution profiles at 45 and 90 min are not shown, for clarity. Each peak was assigned based on the previous results [12], and electrospray mass spectrometry confirmed this assignment. U denotes the fully unfolded protein. (B) Binding property of IAM-C and IAM-D to nAChR. Monoiodoacetamide was added to the refolding mixture to block free sulfhydryl groups, and the acetamide labeled D form (IAM-D) and C form (IAM-C) were prepared with HPLC. The binding to nAChR was assessed as previously described [31]. white square: N form, triangle:IAM-C, circle: IAM-D. (C) CD spectra of N, D and C forms. A positive peak at approximately 228 nm revealed a micro environment of aromatic residues in the folded structure, and a small negative peak at approximately 216 nm, as well as a large positive peak at approximately 197 nm, showed a beta structure [11]. ─:N form, ----:C form (after correction for 22.1% of N form and 7.1% of D form), ┄┄┄: D form (after correction for 13.8% of N form and 55.3% of C form).

The refolding process of Tox62 was followed by HPLC (Fig 2A), which gave elution profiles of N (native 4 disulfide bonds), C and D forms that were identical to previous results [8,12]. A large difference in the retention times between the native and the refolding species should be noted, as this suggested extensive structural transitions and a lower surface hydrophobicity of the native structure.

A nAChR binding study (Fig 2B) showed native-like binding of the C form (123% of N form) but not for the D form (49% of N form). CD spectra of the folding intermediates (Fig 2C) were obtained for samples that were not treated with monoiodoacetamide, so that the native chemical properties of the sulfhydryls of cysteine residues were conserved. The spectra of C form and the D form were therefore corrected for other oxidized forms based on the HPLC analysis. The presence of native secondary structures in the C form suggested that the disulfide bond S17-S41 had stabilized the hairpin structure of finger 2, while in the D form, instability or a significant difference in the β-turn structure of finger 2 was evident. This result, together with lower binding activity and the retarded elution relative to the C form from HPLC, suggested that the D form had formed some native-like structure but lacked a stable β hairpin structure at finger 2, which is thought to be important for biological activity [36].

**Fig 3:**
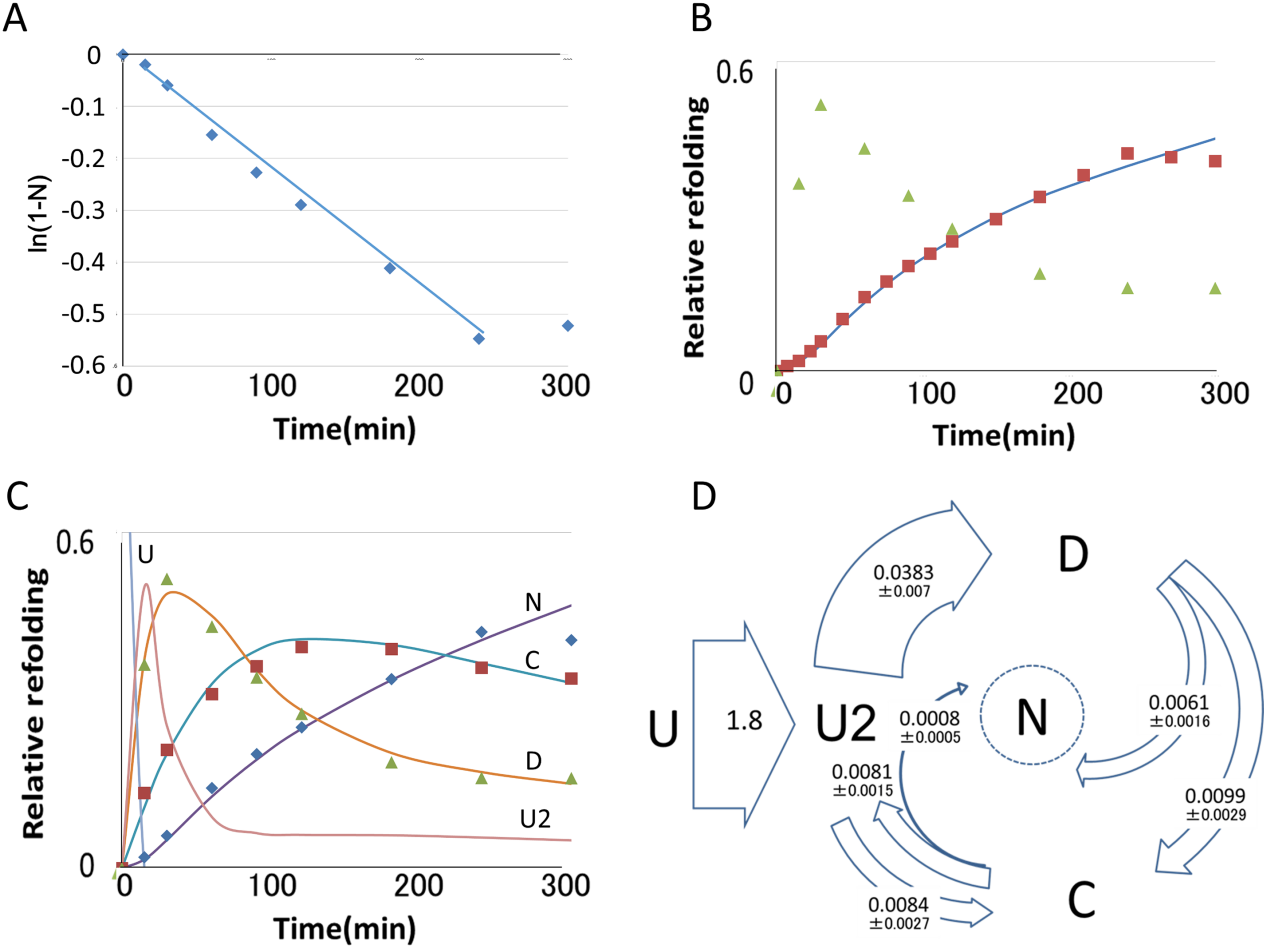
The D form is the immediate precursor to the native form. (A) Exponential decay of unfolded species. The logarithm of 1-N, where N was the species other than the C, D, U (fully unfolded species), and U2 forms (2 disulfide-bonds species), was plotted against time. (B) The D form is the direct precursor to the native form. triangle; observed D, square; observed N and their linear interpolation, ――; calculated N from observed D and their linear interpolation. (C) The validity of the folding model of Tox62 in Fig 3D. The model was assessed using one of the ten refolding datasets for Tox62, by model fitting using Kintecus, [32] to obtain R squared values of 99.6 (N), 99.4 (C) and 99.4 (D). rhombus; observed N, triangle; observed D, square; observed C, each line was calculated by Kintecus. (D) Estimation of relative rate constants of the folding reactions. Ten independent datasets for Tox62 folding were processed independently using Kintecus. Averaged percent R squared values are 99.4 (N), 98.4 (C), and 98.2 (D) when 6 principal reactions were involved in the fitting. The application of the criteria described in the methods section identified reactions other than C to N as indispensable principal reactions because they were essential to maintaining percent R-squared values higher than 98.0, as indexed in the Kintecus fitting. The reactions D to U2 and C to D were eliminated based on the following results. By including these reactions, the rate constants of the 5 principal reactions fluctuated to increase the averaged standard deviations to 1.184 times for D to U2 and 1.384 times for C to D. Pair-wise, one-tailed Student’s t-test assessed the significance of the increase in standard deviations for the rate constants of the principal reactions. The p-values for the reactions D to U2 and C to D were 0.031 and 0.029, respectively. Reaction C to N was maintained, due to the unaltered average of the standard deviation (1.007 times), low significance (p = 0.235) in the t-test, and negligible contribution to the overall reactions.

The refolding of Tox62 proceeded at a rate constant of 4.5 x 10^−5^ s^-1^ for all the unfolded intermediates, provided that the concentration of oxidized glutathione remained constant (Fig 3A). This refolding property was shared with the mutants, except for the rate constants. In Fig 3D and S1 Table, it can be observed that the U2 (primarily in the form of a two disulfide bond species containing S3-S24 and S55-S60), D, and C forms became the major intermediates after a very short period of lag time. The rate constants of each reaction suggested that the rate-limiting step was the D form to N form, while the U2 and C forms served as a reservoir for the D form. The D form introduced irreversibility in the recursive refolding process. It had previously been shown that Toxin α, which has a shorter Lk1 chain than Tox62, by one residue, also folded using the D form as an immediate precursor [10]. The local secondary structures of a long Lk1 should have some mechanisms to stabilize the disulfide bond S43-S54, to separate S41 from S17, and to ultimately stabilize the D form. The C form was not productive, in spite of its stable and native-like structure. This result suggested that the disulfide bond S43-S54 was essential for a production process.

### Absence of native correlations among turns

Table 2 and S2 Table show that YNGK stimulated folding at active turns (turns 2 and 4) and delayed folding at passive turns (turns 1 and 3). Stimulated folding by QAAQ at turns 2 and 4 (Table 3) was presumably due to the isomerization of proline residues at these turns in the native structure. The refolding rates of mutants with multiple mutations were practically identical to the values calculated as multiplication products of the folding rates of single mutants (Table 2). For instance, the observed folding rate of mutant 17 (2.28) was close to the product of the folding rates of mutants 1, 3, and 4 (0.66 x 0.64 x 4.46 = 1.88). The calculated contributions of QAAQ and FDGQ at their respective turns to the folding rate of the mutants supported this observation as a general rule (Table 3 and S2B and S2C Table).

**Table 2:** Folding rates of mutants.

mutant	turn 1	turn 2	turn 3	turn 4	observed folding rate	calculated folding rate
1	YNGK				0.66	
2		YNGK			8.79	
3			YNGK		0.64	
4				YNGK	4.46	
5	YNGK		FDGQ	QAAQ	1.10	1.19
7	QAAQ		FDGQ	YNGK	1.88	2.06
11	QAAQ		QAAQ	QAAQ	1.74	1.84
12	YNGK		QAAQ	QAAQ	1.67	1.73
13	QAAQ		YNGK	QAAQ	1.33	1.23
14	QAAQ		QAAQ	YNGK	3.05	2.99
15	QAAQ	QAAQ	QAAQ	QAAQ	4.64	5.30
16		QAAQ			2.88	
17	YNGK		YNGK	YNGK	2.28	1.88
60		PG			22.59	
Tox62	QSSQ	SPGE	DHRG	KPGI	1	

The refolding rates of mutants were obtained as in Fig 3A and are summarized as rates relative to the Tox62 folding rate obtained by a parallel experiment. Data for the N form, within the range of 8% and 85% of fully folded results, were used, with one exception for mutant 60, where the N form was enriched to 90.7% after 60 min of refolding. Averaged R squared value of the linear regression was 0.99 ±0.013 when mutant 60 (R squared value of 0.786) was excluded. The values in the last column are the multiplication products of the values in Table 3, which confirm the absence of correlations among distant local sequences at the rate-limiting step.

**Table 3:** Absence of mutual correlations among local structures at the rate limiting step.

mutation	turn 1	turn 2	turn 3	turn 4
PG		22.6		
QAAQ	*0*.*70± 0*.*11(4)*	2.88	*0*.*95± 0*.*16(4)*	*2*.*75± 0*.*35(5)*
YNGK	0.66	8.79	0.64	4.46
FDGQ			*0*.*66±0*.*03(4)*	

Relative contributions of mutations to the folding rate. Values in italics are calculated from the observed data in Table 2 on the assumption that mutations contribute to the folding rate in the absence of any mutual correlations among sequences at other turns. The number of calculations for each value is given in parentheses.

### Active turns were involved in global movement

**Fig 4:**
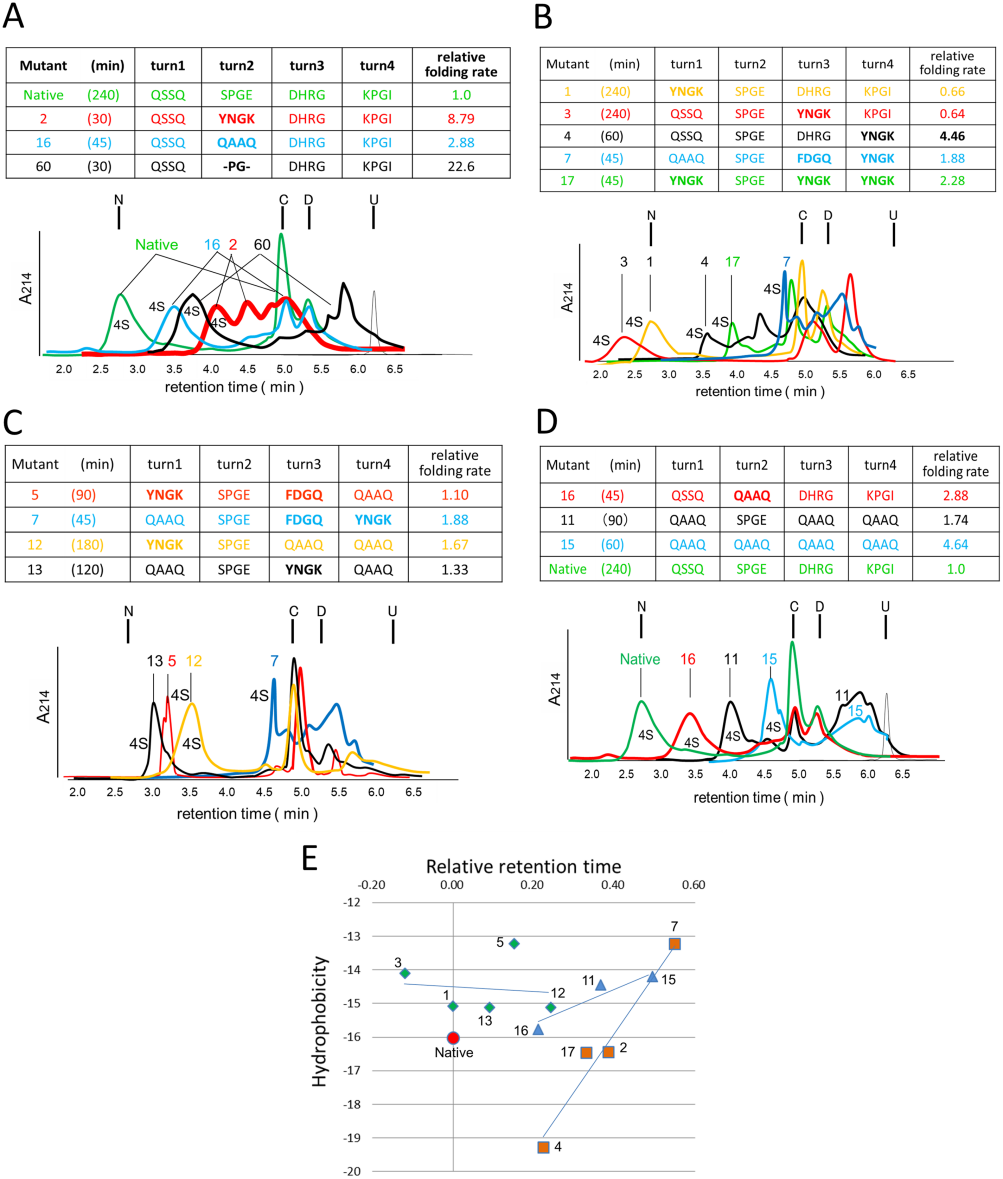
Interpretation of the elution profiles of mutants on HPLC. Labels N, C, D, and U on top of the elution profiles represent the averaged retention times of these respective species. Numbers in the parentheses in the first column are the sampling times after the initiation of folding reactions. Relative hydrophobicity was calculated as the sum of the hydrophobicity of four tetra-peptides at turns (Table 1). (A) Rigid YNGK at turn 2 delayed the elution of 4S species. (B) YNGK at turn 4 introduced analogous consequences as at turn 2. In contrast, YNGK at turn 3 (but much less so at turn 1) accelerated the elution of 4S species. This contribution of YNGK was suppressed in mutant 17 by YNGK at turn 4 (Fig 4E). (C) Comparison of retention times among mutants with the same sums of hydrophobicity. Mutants 5 and 7 and mutants 12 and 13 had equal sums of hydrophobicity. The contribution of YNGK at turns 3 and 4 to the retention time was greater than that of YNGK at turn 1. (D) Absence of YNGK restored the normal correlation between the retention time and the summation of the hydrophobicity of four turns. (E) Summary of the effect of YNGK on retention time. The species without YNGK (mutants 11, 15, 16 and native) form a rough border in the diagram to separate short-retention time mutants in the upper-left region and long-retention time mutants in the lower-right region. Linearity between the hydrophobicity and the retention time was assessed for three groups of mutants. red square: Mutants with YNGK at turns 2 and 4 (mutants 2, 4, 15, and 17), R^2^ = 0.967 (linear regression); green triangle: Mutants with YNGK at turns 1 and 3 but not 2 or 4 (mutants 1, 3, 5, 12, and 13), R^2^ = 0.011 (linear regression); blue triangle: Mutants without YNGK (mutants 11, 15, 16), R^2^ = 0.901 (linear regression); red circle: Native (Tox62).

Fig 4A and 4B show that YNGK at the active turns (turns 2 and 4) delayed elution of 4S (four disulfide bonds) species on HPLC, while elution was accelerated or unaffected by the YNGK at the passive turns (turns 1 and 3). The total hydrophobicity of the four turns does not account for these results (Table 1). In mutant 17, the contribution of YNGK observed at turn 3 in mutant 3 was suppressed by the presence of YNGK at turn 4 (Fig 4B and 4E). This suppression is an example of the emergence of mutual correlations among local structures in 4S species that was absent in Table 2. These results, together with others, are summarized in Fig 4E and S3 Table. In this diagram, the contribution of YNGK at active turns to delay elution appears to be significant (mutants 4, 2, 17, and 7). This contribution should be, at least in part, the consequence of the rigid physical property of YNGK, as the proteins that are frozen in movement should hold water molecules as if in a frozen state, creating an environment analogous to a hydrophobic environment. The opportunities for an internal hydrophobic moiety to be exposed would also increase. Therefore, the consequence of frozen movement should be more significant for proteins with higher surface hydrophilicity. As is shown in Fig 4E, the hydrophilic property of the surface area of mutant 4 was more significant than that of other mutants with YNGK at active turns (mutants 2, 17, and 7), and, as expected, the decrease in the hydrophilic property of mutant 4 was the largest among the tested mutants.

The retardation of the elution on HPLC suggested the rigid structure of YNGK at the active turns froze free global movements of local structures in 4S species. In other words, for the 4S species to have native-like structural property, flexible movements at the active turns was essential. The formation of the disulfide bond S17-S41 as the 4th disulfide bond had been stimulated by YNGK at the active turns, while YNGK at the passive turns had slightly delayed the same process. After the formation of the 4th disulfide bond, the rigid YNGK at the passive turns tended to help form a compact structure, while YNGK at the active turns impaired the native-like structural property. The rigid structure of YNGK thus delineated drastic changes in the role of active turns and passive turns. As described and confirmed below, such changes revealed the distinct assembly and activation processes. The contribution of YNGK at turn 1 was only marginal in retention time, suggesting function of finger 1 was different from that of the finger 2, another finger with passive turn 3.

### Evaluation of the correlations of observations

**Fig 5:**
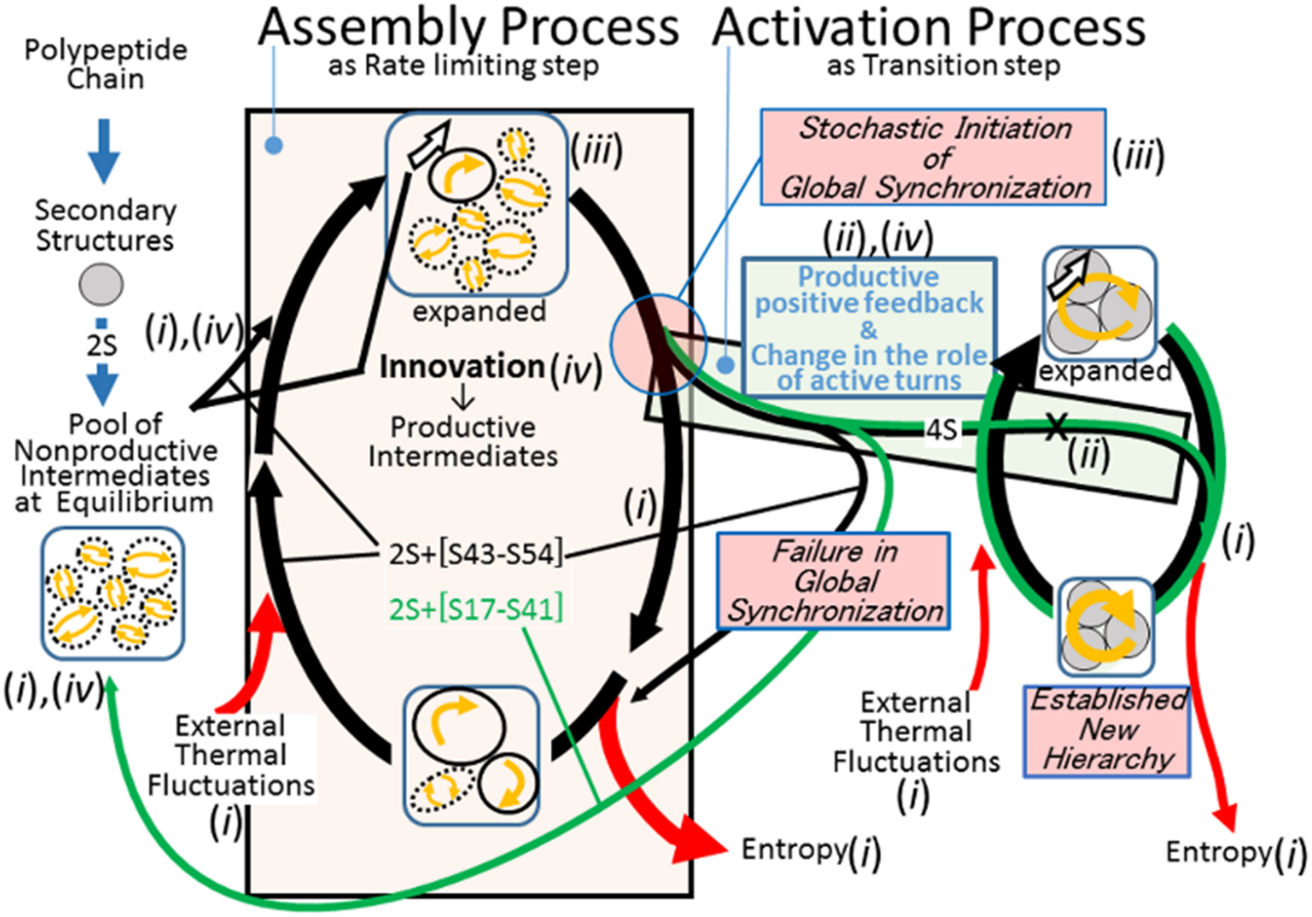
Proposed folding model. In the earlier stages of the folding, each intermediate is essentially formed as a cluster of locally consistent secondary structures. The absence of native correlations among the local structures results in an equilibrium state of the intermediates. Therefore, the intermediates are saturated with entropy and the formation of S43-S54 as an innovation provides the means to address this entropy. In the assembly process, the rate-limiting stochastic process searches for a productive combination of local structures in terms of relative orientations and synchronization. Positive feedback processes initiate the activation process, and the successful propagation optimizes and establishes the global synchronization of secondary structures for efficient entropy release. The efficiency of entropy release can be estimated as entropy release per unit of time. The number of steps per cycle can represent time per cycle, which is smaller for the compact native state, where S17-S41 further restricts alternative movements. Furthermore, a logarithm of the number of steps can be proportional to the entropy release per cycle. This assumption is based on the expectation that, in the process to load the external thermal fluctuation in local structures, such as finger 3, the number of possible structural configurations at step n could be proportional to n, provided that the surface area to accept the external thermal fluctuation is not altered. In other words, the probability to choose the current configuration is proportional to 1/n. Then, ln (n), which is the integral of 1/n, measures the accumulated difficulty to reverse the whole process back to the initial state. Therefore, ln(x1)/x1 > ln(x2)/x2, where x1, the number of steps in the cycle of native form, < x2, the number of steps in the cycle of the D form. It is noteworthy that x1 cannot be shorter than the possible cycle time of the synchronized global movements. *(i)-(iv)*, critical evidence listed in the text to support the respective states or steps in this model; blue arrow, processes for 2S (S3-S24, S55-S60) species formation; black arrow, processes where 2S and S43-S54 are involved; green arrow, processes where 2S and S17-S41 are involved; box, intermediates or folded native protein; curved white arrow, disulfide bond S43-S54 as the key architecture that drives the process to release entropy; arrow in dashed circle, locally closed, self-consistent equilibrium in the movements of atoms or the flow of the physical state in various forms; dashed circle represents stochastic and flexible feature in combination with the secondary structures and the absence of correlations within the pseudo-external environment (see text); arrow in circle, the asymmetric movement of atoms or the flow of the physical state in various forms, without a self-consistent property because of the inevitable external input or output; cyclic arrow, recursive and irreversible cycle of the state of molecules, where the movements of the local atoms are globally synchronized, forming a chain of numerous local circles.

The data obtained thus far suggest a new perspective, in which entropy release to the external environment supports protein folding and the established structure. Pieces of evidence critical to such a perspective are listed below in italics. Their interpretations are summarized in Fig 5.

(i) *The folding process formed a recursive cycle that was practically irreversible* (Fig 3D).
(ii) *In contrast to the stimulated 4th disulfide bond formation, rigid YNGK at active turns delayed the elution of 4S species on HPLC, suggesting the frozen global movement* (Fig 4). *Rigid YNGK at the passive turns returned small but opposite results*.
(iii) *A total absence of native correlations was evident at the moment of the 4th disulfide bond formation* (Table 2). Essentially, the same observations have been reported for a large number of proteins, such as “the transition state ensemble at zero stability is a broad conformational ensemble stabilized by local interactions and without specific tertiary interactions” [37].
(iv) *The most native-like C form was not productive. Furthermore, the D form was not derived from the C form* (Fig 3D). *The disulfide bond S43-S54 uniquely emerged in the D form, suggesting that foldability arose from this disulfide bond*.

The recursive behaviour of the intermediates in observation *(i)* was indicative of a secondary role for Gibbs free energy in folding. The possibility that the folding species are equipped with some mechanism to break the equilibrium was also suggested. Furthermore, according to classical thermodynamics, a recursive and irreversible process means the repetitive production and release of entropy. Observation *(i)* should therefore be interpreted to indicate that the external perturbations provided thermal fluctuations, which were transformed and released to the external environment as entropy. For entropy to be a major player, there had to be some facilitation in the structure. The possible facilitation will be investigated in the next section.

The observation *(ii)* revealed that the flexible movement at the active turns played a crucial role in supporting global movement in the 4S species; therefore the impaired movement by a rigid YNGK extensively delayed the elution of 4S species on HPLC. Obviously, to squeeze out entropy efficiently, it should have been required that no room remain inside that may accommodate residual entropy. In the succeeding step, the structure would be relaxed to accept the external thermal perturbation. The globally synchronized movement to switch to either a compressed or a relaxed state, in turn, should maintain the system in a non-equilibrium state. As will be described later, in the presence of an inevitable asymmetric flow of the physical fluctuations, the globally synchronized structural movements, compact structure formation, and entropy release support each other to form a positive feedback process.

The observations *(ii)* and *(iii)* revealed two distinct processes, namely, the assembly and activation processes. The assembly process is a process to search for productive combinations of native-like secondary structures in the absence of native correlations. As will be described below, this stochastic process should be a general process to establish a new hierarchy. Rigid YNGK at the active turns accelerated the assembly process, presumably by reducing both the search space and the opportunities for disulfide bond cleavage. The activation process is a process for the propagation of positive feedback, which is interrupted by a reduction in global structural movement. For the initiation of the activation process, it was highly noteworthy that all the local structures were at their correct positions, similar to pieces of a jigsaw puzzle, so that the explosive buildup of global native collaborations towards a unique answer could be triggered. In other words, if the local structures of the folding molecules had multiple possibilities, the folding would fail to proceed to the activation process. This activation process was substantially comparable to a card game where a player won when a set of cards formed a unique competitive meaning. An exclusive entrance or down-hill funnel as a map of a single structure was established at this moment. The number of value sets, as shown in Table3, resulted in a number of differently folded structures. Because the set of the values in Table3 was one, the native-folded structure was essentially shared among all the mutants used in this study.

Observation *(iv)* was enough to identify the disulfide bond S43-S54 as pivotal architecture for the release of entropy to the external environment. Due to the absence of the disulfide bond S43-S54, the C and U2 forms could not address external thermal fluctuations. Therefore, they remain saturated with entropy. As the observation *(ii)* revealed, the formation of the 4th disulfide bond was an indication of the successful assembly process and a signal to proceed to the activation process. The globally synchronized movements could stabilize the disulfide bond S17-S41, and this disulfide bond allowed the molecular-wide global synchronization, which was absent both in the D form and at the moment of 4S species formation. The movements could ensure the successful activation process this way. In the 4S species, the disulfide bond S43-S54 contributes to entropy release and the disulfide bond S17-S41 contributes to molecular-wide synchronization. Therefore, the elements that support entropy release and the synchronized molecular-wide movements wait for the combination of structures to form a positive feedback process. This positive feedback process operated between the efficiency in entropy release and the optimized and synchronized movements of the well-packed, compact, secondary structures. The synchronized movements of all the local structures resulted in the emergence of a new function to release entropy. In general, if a certain combination of the elements results in a new function, such as entropy release, the elements, such as the disulfide bonds S17-S41 and S43-S54, belong to a lower hierarchy. The higher hierarchy is characterized by the new function. This relationship is propagated and stabilized when the positive feedback is operative between the two hierarchies. The relationship between the elements for combination in the lower hierarchy and the function of the higher hierarchy could also be expressed by a logarithmic function. Even though the ultimate reason for the emergence of biological systems as a dissipative structure was not necessarily clarified, the folding system could decrease the unpredictable and inevitable interference from the external environment through the creation of an internal flow of entropy as a pseudo-external environment. This creation of entropy flow is analogous to Benard’s cells, where the well-organized movement of water molecules efficiently release the internal thermal fluctuations that could otherwise accumulate. How could all of this be accomplished? The native structure should have the answer.

### Verification of the observations in the native structure

**Fig 6:**
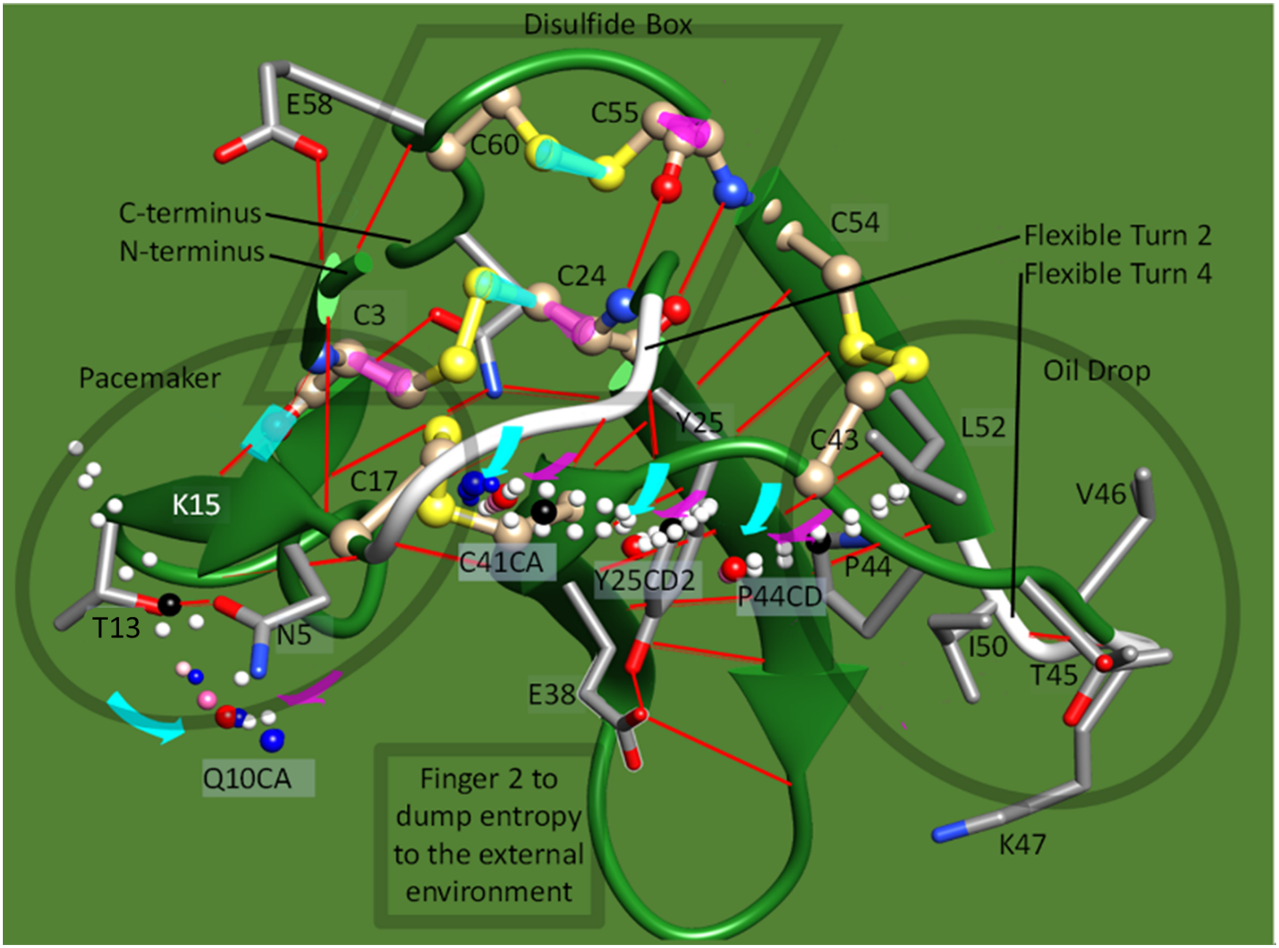
Simulation of synchronized oscillation in 5ebx.pdb. The calculated locations of the α carbon atoms for Q10 (Q10CA) and C41 (C41CA), as well as for the CD2 atom of Y25 (Y25CD2) and the CD atom of P44 (P44CD), were superimposed as isolated balls on the structure of 5ebx.pdb. The red, pink, or white balls for the last three atoms are shown at the simulated locations for the oscillation range of ±10 degrees from the original positions, which are shown as black balls. The cyan and magenta rods drawn on the bonds are the axes of rotation. The apparent locational deviation of some black balls from the 5ebx.pdb structure was due to artificial data processing in the graphical tool to draw the 5ebx.pdb structure smoothly. The big red or big blue balls are at the positions where the rotations along the magenta axes are at the maximum (+10 degree), while the pink balls show the next locations to visit. Curved magenta and cyan arrows show the rotation of the atoms approaching the positions of the respective big red or big blue balls. Blue balls show the coordinates when the CD of P44 moves as shown in the figure, without limitation from CD2 of Y25 by van der Waals contacts. In contrast, we assumed that the CD of P44 hits the CD2 of Y25 to trigger the formation of a soliton wave (see text for details). It was postulated that the phase of rotation along the cyan rods, except for the one built with K15 and C3, was preceded by rotations along the magenta rods. This delay was to take into account a correlation that the approaching P44 forced the swing along the cyan rods described above. Additionally, the flexible feature of turn 4 should not have transmitted the physical movement of the latter half of finger 3 (such as peptide 50–55) to the floppy peptide chain 41–49 directly. In this simulation, the postulated delay in phase was 45 degrees.

**Fig 7:**
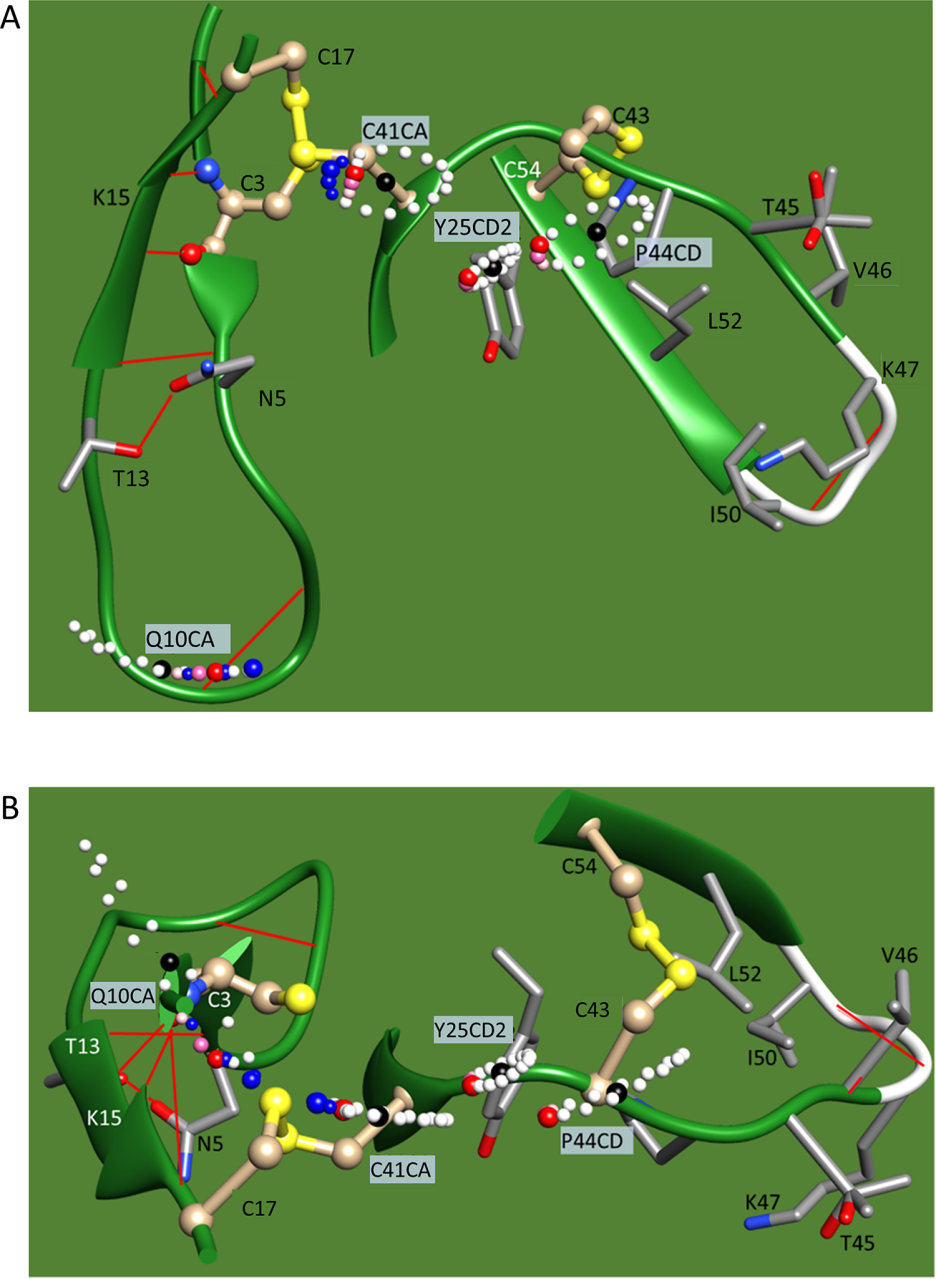
Side and top views of the simulation of a synchronized oscillation in 5ebx.pdb. (A) Side view, (B) Top view. Figure notations are the same as in Fig 6.

In the native structure, the disulfide bond S43-S54 guided the movement of the peptide chain 41–49 in finger 3 (Figs 6 and 7 and S4 Table), so that the side chain of P44 in finger 3 collided with the side chain of Y25 on finger 2. In the simulation in Figs 6 and 7, rotations along the magenta rods in Fig 6 bring the side chain of P44 closer to Y25, driving the rotation of the cyan axes, except the one formed between K15 and C3. The simulated distance between the CD2 atom in Y25 and the CD atom in P44 was thus found to deviate by approximately 1 Å from the value of van der Waals contacts in the reported structure (varied from 3.45 Å to 2.46 Å and 4.47 Å, respectively). By this collision, an inertial moment in the floppy side chains in the “oil drop” of finger 3 was transferred to the peptide chain and transmitted towards C41 in the form of a soliton [38], where various bond angles and bond length were strained and relaxed. The formation of the hairpin structure of finger 2 should be encouraged because R39 in the turn configuration was extended, and the guanidine group of its side chain was placed towards the C-terminal carboxyl group. Furthermore, the heavy sulfur atom S41 was brought closer to sulfur S17 of C17 to form a disulfide bond. Then, the firm finger 2 should be the better destination of a soliton wave for its rigid and peripheral features.

The swirling oscillation of finger 3 could be mainly supported by rotation axes of magenta and cyan rods on C55 and SG atom of C60 (Fig 6). Collision of P44 in finger 3 with Y25 in finger 2 would then force movement of the finger 2 along rotation axes such as magenta and cyan rods on C24 (Fig 6). Balls in Figs 6 and 7 show the locations of some critical atoms moving along their respective axes for ±10 degrees from the published coordinates. Such a large angle could be an exaggeration, especially for Y25, due to its physical environment at the central core structure, but they should be optimized during the folding process. P44CD and C41CA rotated along the C55 axis, while Y25CD2 rotated along the C24 axis. In this simulation, P44 with a longer rotation radius approached Y25 beyond the van der Waals radii, so that Y25 worked as a physical limiter of the swirling oscillation of the floppy peptide chain 41–47. The simulated locations of C41CA and Q10CA were calculated based on the assumption that interatomic distances between the side chains of P44 and Y25 at collision should not be shorter than 3 Å. As was shown in Fig 6, the trajectory of Q10CA did not fluctuate by this restriction when compared to the ones where van der Waals restrictions were ignored (blue balls). Some others, including flexible active turns 2 and 4, also contributed to the behavior of the atoms, but the presence of disulfide bonds S43-S54 and S3-S24 should have rendered their effect secondary.

This simulation further revealed that the N-terminal domain formed a cluster to work similar to a pacemaker, such that turn 2 in Lk1 transmitted the swinging movement of the cluster to the strand central to the whole molecule, to modulate the synchronization. A longer Lk1 is likely to have reduced modulating function.

Butterfly-like periodic molecular movement of expansion and compression emerged as a consequence of simulation to support the validity of the proposed folding model, in which globally synchronized movements of the structure efficiently release entropy that has derived from the external thermal fluctuations, and motivation for releasing the entropy drives the protein folding and maintains the native structure.

## Discussion

The results of this study suggested that protein folding proceeded in a two-step process. In a single-step model, the assembly and activation processes could proceed in parallel. The results in Table 2 denied this possibility because the native-like mutual relationships among secondary structures were not formed by the time of 4S species formation. In other words, the native-like secondary structure formation proceeded without any support from the native-like molecular-wide mutual correlations, while the formation of the native-like mutual correlations among the secondary structures had to wait for the 4S species formation. Therefore, the results in Table 2 suggested a two-step folding process, where the native-like secondary structures were prepared so that various combinations of secondary structures could be evaluated in the assembly process to determine whether to proceed to the second step of the activation process. Native, molecular-wide, mutual correlations were established in this activation process. For the foldability of the proteins, a secondary structure with the capability to drive entropy release, similar to the disulfide bond S43-S54, should be present as a member of the productive combination. For the fast folding proteins, this rate-limiting assembly process could have been overlooked. The use of YNGK at the active turns allows for the freezing and visualization of such a rapid process.

According to the previous theoretical studies on the dissipative systems [39], Poincare resonance introduces mutual correlations that are the origin of irreversibility. In this study, a molecular-wide global synchronization of local structures was suggested to establish the inherent properties of the native proteins where all the local structures are mutually acknowledged. Turns 2 and 4 are located at positions where their close-open movements are closely conjugated with changes in the distances among local structures. Therefore, the consequences of the presence of the rigid YNGK, such as the ones in Fig 4, should be understood primarily as the consequences of decreased movements for the 4S species. Furthermore, the random motions of local structures result in the equilibrium state, where the internal entropy is saturated and entropy release is not expected. As confirmed in Fig 6, the innovative formation of the disulfide bond S43-S54 also denied random movement. Therefore, among the two modes of the possible movements, synchronized movement remains the only movement. The synchronization and the resonance are analogous, as some physical features that permit interactive communications are shared among the elements.

The creation of a new hierarchy is highlighted in this study. This creation is different from the ordinary phase transition, as a stochastic but productive combination of the elements in the lower hierarchy triggered the creation of a new hierarchy that functioned to support the state of the lower hierarchy. Positive feedback propagated and established the states of the two hierarchies this way. Before the creation of the new hierarchy, the mutual ignorance among the secondary structures was demonstrated in Table 2. This mutual ignorance among the elements is a general requirement for the explosive progress of the positive feedback and for the stability of the established hierarchies. Creativity should also be a crucial feature of this process. The molecular-wide global synchronization was permitted only after the 4th disulfide bond was formed and communicative relationships among the secondary structures in the native protein could be established. The assembly process was a stochastic process to explore the productive combination of the moving secondary structures, while positive feedback between synchronization and entropy release was initiated and propagated in the activation process to create a higher hierarchy. Then, the synchronized movements of the local secondary structures occurred in the lower hierarchy, while in the higher hierarchy, the repeating flow and release of thermal fluctuations occurred. This flow of the thermal fluctuations was driven by an inertial moment, provided by the collision of the synchronizing secondary structures in the lower hierarchy. The floppy thermal fluctuations in the oil-drop-like cluster of atoms in finger 3 were thus displaced towards finger 2 in the form of a soliton wave [38]. It is noteworthy that, even though the kinetic energy in the lower hierarchy was responsible for the inertial moment in the higher hierarchy, the kinetic energy of the Brownian movements themselves remained shared in the lower hierarchy. In other words, kinetic energy was not generated in the higher hierarchy because this soliton wave was merely a flow of the thermal fluctuations driven by the inertial moment. However, the periodic property of the soliton wave or its modulation can be informative to express the physical state both internally and externally. Because the structure is shared among the hierarchies, the amount as well as the mode of entropy release from the higher hierarchy and the synchronized movements of secondary structures in the lower hierarchy are mutually linked by positive feedback to establish efficient entropy release. The term pseud-external environment is used to describe the attributes of the higher hierarchy because the flow of the entropy in the higher hierarchy provides an environment where the local structures in the lower hierarchy inhabit forming a community. The thermal fluctuations in the local structures were irreversibly displaced to release entropy to the external environment. This process is compatible with the irreversible displacement of heat energy between two heat reservoirs of different temperatures. The applicability of classical thermodynamics to predict the goal of the folding process is limited, however, because the stochastic combination of the elements in the lower hierarchy and the positive feedback create the higher hierarchy.

Finally, the applicability of the proposed protein folding mechanism to ordinary, non-disulfide bonded proteins has to be argued. In this respect, it is noteworthy that the state of the D form could have been a native state if the local structure, including the additional residues in long Lk1, evolved (or even degenerated) to maintain the peptide chain 41–43 close enough to Lk1. In this case, the disulfide bond S43-S54 is not essential to the critical folding processes. Hydrophobic amino acids may replace C17 and C41 as long as robustness is not an issue. The disulfide bond could be a choice, unless a significant number of amino acid residues are available to establish the equivalent function. We therefore tentatively propose that the folding mechanism revealed in this study could hold for non-disulfide bonded proteins. Obviously, further investigations are required using many other proteins.

## Conclusion

Protein folding can be described as a process to create a new hierarchy that functions to release entropy efficiently. Protein folding is motivated by a need to release the inevitably accumulated internal thermal fluctuations to the external environment. The release of the internal thermal fluctuations is equivalent to the release of internal entropy. In the rate-limiting assembly process, all the secondary structures as candidate members of the lower hierarchy were waiting for combination and behaved without any native mutual collaboration. This mutual ignorance enabled stochastic exploration for the correct combination of moving secondary structures. In the activation process, the higher hierarchy was established by the collaboration of the secondary structures in the selected combination, which had been evaluated in the assembly process. This occurs in the lower hierarchy, while the new function belongs to the new higher hierarchy. The mutual ignorance among the secondary structures in the assembly process was all of a sudden replaced by native collaborations, which are mutually supportive with molecular-wide global synchronization. The synchronized Brownian movements in the lower hierarchy provided the inertial moment. In the higher hierarchy, this inertial moment drove the displacement of thermal fluctuations in the cluster of atoms such as in finger 3. The thermal fluctuations were thus released in the form of a soliton wave. The positive feedback propagated and stabilized both the function of the higher hierarchy and the selected combination of the secondary structures in the lower hierarchy. The formation of a compact and flexible structure and the efficient entropy release were thus established.

## Supporting information

S1 Table Evaluation of the possible folding reactions and rate constants for ten folding datasets of native protein. A: Relative amounts of N, C, and D forms as analyzed by Kintecus. B: Candidate reactions were eliminated when t-tests revealed significant increases in the standard deviations of the rate constants. Before the t-tests, the standard deviations were normalized to compensate for the differences in the averaged rate constants.
(XLSX)

S2 Table The absence of mutual correlations among turns at the rate-limiting step. A: The folding rates of mutants were calculated from the observed N forms. The logarithms of 1-N, where N was the ratio of folded protein to total protein, were plotted against time to calculate the relative folding rate. Data representing 8% to 85% folded protein were used exclusively. Some data were further eliminated from the calculations for reasons described in the Table. Data used in the calculations are colored red. B: The observed folding rates were compared to their calculated folding rates. C: Calculations of the parameters P2, P3, P4, and P10. D: Summary of the parameters that were used in Table B to calculate the folding rates of mutants.
(XLSX)

S3 Table Correlation between relative retention time on HPLC and hydrophobicity of turns. A: Relative retention times of 4S species and the sum of the hydrophobicity of four turns. Tables B, C, and D were used to calculate linear correlations.
(XLSX)

S4 Table Axes of rotations and the calculated coordinates of atoms. The axes of rotations, angles of rotations, and delay in the phase of rotations should have been optimized for the increased efficiency of entropic release. In this simulation, the first and the second axes were essentially perpendicular to each other, and the change in the angles was ±10 degrees. The delay in the phase of the second axis was 45 degrees. The blue cells in the table present the data from the blue balls in Figs 6 and 7, which are expected in the absence of the physical limitations caused by van der Waals contacts between atoms in P44 and Y25.
(XLSX)
